# Molecular and Phenotypic Characterisation of Paediatric Glioma Cell Lines as Models for Preclinical Drug Development

**DOI:** 10.1371/journal.pone.0005209

**Published:** 2009-04-14

**Authors:** Dorine A. Bax, Suzanne E. Little, Nathalie Gaspar, Lara Perryman, Lynley Marshall, Marta Viana-Pereira, Tania A. Jones, Richard D. Williams, Anita Grigoriadis, Gilles Vassal, Paul Workman, Denise Sheer, Rui M. Reis, Andrew D. J. Pearson, Darren Hargrave, Chris Jones

**Affiliations:** 1 Paediatric Oncology, The Institute of Cancer Research, Sutton, United Kingdom; 2 Cancer Research UK Centre for Cancer Therapeutics, The Institute of Cancer Research, Sutton, United Kingdom; 3 Pharmacology and New Treatments of Cancer, Institut de Cancérologie Gustave Roussy, Villejuif, France; 4 Paediatric Oncology, The Royal Marsden NHS Foundation Trust, Sutton, United Kingdom; 5 Life and Health Science Research Institute (ICVS), Universidade do Minho, Braga, Portugal; 6 Neuroscience Centre, Institute of Cell and Molecular Science, Bart's and The London School of Medicine & Dentistry, London, United Kingdom; 7 Breakthrough Breast Cancer Unit, Guy's Hospital, London, United Kingdom; Ordway Research Institute, United States of America

## Abstract

**Background:**

Although paediatric high grade gliomas resemble their adult counterparts in many ways, there appear to be distinct clinical and biological differences. One important factor hampering the development of new targeted therapies is the relative lack of cell lines derived from childhood glioma patients, as it is unclear whether the well-established adult lines commonly used are representative of the underlying molecular genetics of childhood tumours. We have carried out a detailed molecular and phenotypic characterisation of a series of paediatric high grade glioma cell lines in comparison to routinely used adult lines.

**Principal Findings:**

All lines proliferate as adherent monolayers and express glial markers. Copy number profiling revealed complex genomes including amplification and deletions of genes known to be pivotal in core glioblastoma signalling pathways. Expression profiling identified 93 differentially expressed genes which were able to distinguish between the adult and paediatric high grade cell lines, including a number of kinases and co-ordinated sets of genes associated with DNA integrity and the immune response.

**Significance:**

These data demonstrate that glioma cell lines derived from paediatric patients show key molecular differences to those from adults, some of which are well known, whilst others may provide novel targets for evaluation in primary tumours. We thus provide the rationale and demonstrate the practicability of using paediatric glioma cell lines for preclinical and mechanistic studies.

## Introduction

Paediatric high grade glioma is a clinically devastating and biologically under-studied cancer of the central nervous system. The poor clinical outcome of these children is largely due to the intrinsic drug resistance of these tumours coupled with a lack of understanding of the basic molecular pathology of the disease [1].

Unlike adults, little is known about the mechanisms of tumorigenesis of glioblastoma in children. *TP53* mutation and PDGFRα overexpression, alterations associated with secondary glioblastoma in adults, have been reported at high frequencies in paediatric cases, [2], however these tumours rarely originate from pre-existing low grade lesions [3]. Furthermore, although there are clear morphological similarities between high grade gliomas of all ages, the WHO classification system is based upon adult cases, and may not be truly representative of the histopathological diversity of the childhood disease [4]. Firm conclusions are hampered by the limited number of studies performed on small sample sizes.

Drug treatment efficacy also differs. In adult glioblastoma, introduction of combined chemoradiotherapy of concomitant and adjuvant temozolomide (TMZ) and radiotherapy has provided a modest survival benefit [5], particularly in patients with an epigenetically silenced O^6^-methylguanine-DNA-methyltransferase (*MGMT*) gene [6]. In children, hypermethylated *MGMT* promoter predicts for response to alkylating agents [7], however the survival of children treated with adjuvant temozolomide does not appear to be improved when compared with historical controls [8]–[11]. Similarly, the disappointing results obtained in early phase clinical trials by the use of inhibitors directed against EGFR [12] and PDGFR [13] are likely in part due to inadequate patient selection, although as yet there are no validated markers predictive of response in high grade gliomas, and it is unclear whether there are differences in the paediatric setting.

The mechanisms of drug resistance in paediatric high grade glioma are poorly understood, in part due to the limited availability of suitable models of the disease. Nearly all preclinical and functional work is carried out in a relatively small panel of adult glioblastoma cell lines *in vitro*, and it is currently unclear how representative these are of the underlying biology of paediatric lesions.

In order to begin to address these questions, we have carried out a detailed molecular and phenotypic characterisation of a series of paediatric high grade glioma cell lines in comparison to routinely used adult lines. Through an integrated profiling approach, we demonstrate that glioma cell lines derived from paediatric patients show key molecular differences to those from adults, some of which are well known, whilst others may provide novel targets for evaluation in primary tumours. We thus provide the rationale for using paediatric high grade glioma cell lines for preclinical and mechanistic studies.

## Materials and Methods

### Cell culture

Adult glioblastoma cell lines, A172, LN229, SF268, U87MG, U118MG and U138MG were obtained from ATCC (LGC Promochem, Middlesex, UK). Paediatric glioblastoma KNS42 cells were obtained from the JCRB (Japan Cancer Research Resources) cell bank. Paediatric SF188 cells were kindly provided by Dr Daphne Haas-Kogan (UCSF, San Francisco, CA, USA), whilst UW479, Res259 and Res186 were kindly provided by Dr Michael Bobola (University of Washington, Seattle, WA, USA). They were grown as monolayers in DMEM/F12 Ham's medium+10% FCS in 5% CO_2_.

### Immunohistochemistry

Cells were grown to 70–80% confluency in chambered slides (Nunc, Rochester, NY, USA) fixed in either 4% paraformaldehyde:0.5% TritonX-100 in PBS (GFAP, synaptophysin, vimentin, nestin and CD133) or ice cold methanol (S100). The cells underwent a peroxidase block (Dako, Ely, UK) followed by washes in 0.05%Tween in TBS prior to incubation with primary antibody – GFAP (clone 6F2, 1∶50 dilution, Dako); synaptophysin (SY38, 1∶10, Dako); vimentin (V9, 1∶100, Dako), S100 (B32.1, 1∶20, Abcam, Cambridge, UK), nestin (10C2, 1∶400, Miltenyi Biotech, Bergisch Gladbach, Germany), or CD133 (W6B3C1), 1∶40, Miltenyi Biotech). Staining was visualised by the Envision horseradish peroxidase system (Dako) according to the manufacturer's instructions.

### Genomic analysis

500 ng of DNA was analysed on the Affymetrix GeneChip Human Mapping 250K Nsp and Sty arrays by the UCL Institute of Child Health Gene Microarray Centre, according to the manufacturer's protocol. The raw data were processed and genotypes called using the BRLMM method of the Affymetrix GTYPE package. Copy number ratios were calculated by the Affymetrix CNAT 4 algorithm, using quantile normalisation and a Gaussian smoothing bandwidth of 0.1 Mb. Normal references were provided by public data from an unmatched panel of 30 Caucasian female DNA samples, the Centre d'Etude du Polymorphisme Humain (CEPH) trio mothers, analysed on the same platform by The International HapMap Consortium (International HapMap Consortium 2007, http://www.hapmap.org). The copy number data set was compared to the positions of known copy number variations (CNVs) in the HapMap populations displayed on the UCSC genome browser and any copy number changes in the current data that overlapped with these CNVs were noted. LOH was inferred using the linkage disequilibrium Hidden Markov Model as implemented in dChip (http://www.dchip.org). False positives were reduced by excluding apparent LOH regions present in more than 10% of all 60 CEPH trio parents. LOH was taken as copy neutral if the copy number measured by dChip was close to diploid (1.7–2.3 copies). All data have been submitted according to MIAME guidelines [14] to the public data repository ArrayExpress (http://www.ebi.ac.uk/arrayexpress/) with accession number E-TABM-579.

Multiplex fluorescence *in situ* hybridisation (M-FISH) analysis was performed on metaphase spreads prepared from Res259 cells using a Vysis SpectraVysion probe (Abbot Molecular, Abbott Park, IL, USA) following the manufacturer's instructions as previously described [15]. Images were captured using a Zeiss Axiophot microscope equipped for epifluorescence using cooled CCD-camera (Hamamatsu, Welwyn Garden City, UK). Image analysis was performed using SmartCapture X software (Digital Scientific, Cambridge, UK).

### Fluorescent in situ hybridisation (FISH)

FISH analysis was carried out on metaphase chromosome preparations as previously described [16]. Probes directed against CDK4 (RP11-66N19,RP11-571M6), PDGFRA (RP11-231C18, RP11-626H4, RP11-117E8), and PTEN (RP11-105A10) were labelled with Cy5 (GE Healthcare, Amersham, UK), whilst commercial probes against RB1 (Abbot Molecular) and CDKN2A/B (Abbott Molecular) were SpectrumOrange-labelled. Appropriate centromeric probes were labelled with either fluorescein or SpectrumGreen. Hybridised preparations counterstained with DAPI in antifade (Vector Laboratories Inc., Burlingame, CA, USA). Images were captured using a cooled charge-coupled device camera (Photometrics, Tuscon, AZ, USA).

### Methylation-specific multiplex ligation-dependent probe amplification (MS-MLPA)

Promoter methylation was assessed by kits ME001, ME002 and ME011 according to manufacturer's instructions (MRC-Holland, Amsterdam, Netherlands) [17]. Briefly, 100 ng of tumour DNA was denatured in 5 µL of Ultra-Pure Water at 98°C for 5 min and then incubated with the probe mix for 20 h at 60°C. After probe hybridization, each sample was divided into two different tubes. Half of the sample was ligated using a ligase enzyme and in the other half ligation was combined with *Hha*I digestion resulting in ligation of the methylated sequences only. The resulting products were amplified by PCR using a FAM-labeled primer, following manufacturer's instructions. PCR products were analyzed on ABI Prism 310 single capillary genetic analyzer (Applied Biosystems) using the GeneScan 3.7 software (Applied Biosystems). Duplicate experiments were performed for methylation analysis and average ratios were calculated. We interpreted ratios as absence of hypermethylation (0.00–0.24), mild hypermethylation (0.25–0.49), moderate hypermethylation (0.50–0.74), and extensive hypermethylation (≥0.75), as previously described [18].

### Western blot analysis

Cells at 80% confluence were trypsinised, washed with PBS and lysed for 1 hr at 4°C in lysis buffer (Cell Signaling, Hertfordshire, UK) and a complete mini protease inhibitor cocktail (Roche Diagnostics, Mannheim, Germany). Cells were then centrifuged at 11,000 rpm at 4°C for 15 min, and protein concentration determined (Pierce BCA assay, Rockford, IL, USA). Total protein extracts (30 µg/lane) were separated electrophoretically in 4–20% SDS-polyacrylamide gel and transferred to nitrocellulose membranes (Invitrogen Ltd, Paisley, UK). Immunodetection was performed using antibodies against cRAF (1∶500, Santa Cruz Biotechnologies, Santa Cruz, CA, USA), phosphorylated and total Erk1/2, Akt, GSK3β and S6 (all 1∶1000, Cell Signaling), and GAPDH (1∶2000, Chemicon, Hampshire, UK). Blots were revealed with peroxidase-conjugated secondary anti-rabbit or anti-mouse antibodies (GE Healthcare, Amersham, UK) followed by ECL chemiluminescence solution (GE Healthcare).

### mRNA expression profiling

10 µg of total RNA was used to generate labelled cRNA and hybridised to U133Plus 2.0 oligonucleotide arrays (Affymetrix, Santa Clara, CA, USA) containing more than 40,000 transcripts by the UCL Institute of Child Health Gene Microarray Centre according to standard manufacturer protocols. Probe intensity data for each array was normalized to a baseline array with median signal intensity using the “invariant set” model. Model-based expression was performed using the perfect-match/mismatch (PM/MM) model to summarize signal intensities for each probe set. All data have been submitted according to MIAME guidelines [14] to the public data repository ArrayExpress (http://www.ebi.ac.uk/arrayexpress/) with accession number E-TABM-579. For the supervised identification of genes exhibiting differential expression between paediatric and adult high grade cell lines, expression values were subjected to a variation filter to exclude probe sets with minimal variation across the samples, and an absolute signal to noise metric of greater than 1.5 was subsequently used to identify marker probe sets using GenePattern software (http://www.broad.mit.edu/cancer/software/genepattern/);. Differentially expressed co-ordinate sets of genes were identified using Gene Set Enrichment Analysis (GSEA, www.broad.mit.edu/gsea/) with a nominal p value cut-off of 0.001.

### Real-time quantitative RT-PCR

cDNA was prepared from 1 µg of tumour or reference RNA by random primed reverse transcription using Superscript II (Invitrogen, Paisley, UK). TaqMan® Gene Expression Assays were obtained from Applied Biosystems (Warrington, UK) for LYN (Hs00176719), CRKL (Hs00178304), AXL (Hs00242357) and EPHA6 (Hs00297133). PCRs were performed in a 10 µl reaction volume containing 5 µl 2× buffer/enzyme mix, 0.5 µl 20× assay mix, 0.5 µl 20× GAPDH endogenous control assay mix (Hs99999905) and 1 µl input cDNA. Assays were run on an Applied Biosystems 7900 Sequence Detection System and results analysed by the standard curve method. Data were normalised to Universal Human Reference RNA (Stratagene, La Jolla, California, USA).

## Results

### Paediatric glioma cell lines grown as monolayers in vitro express astrocytic and stem cell markers

**Figure 1:**
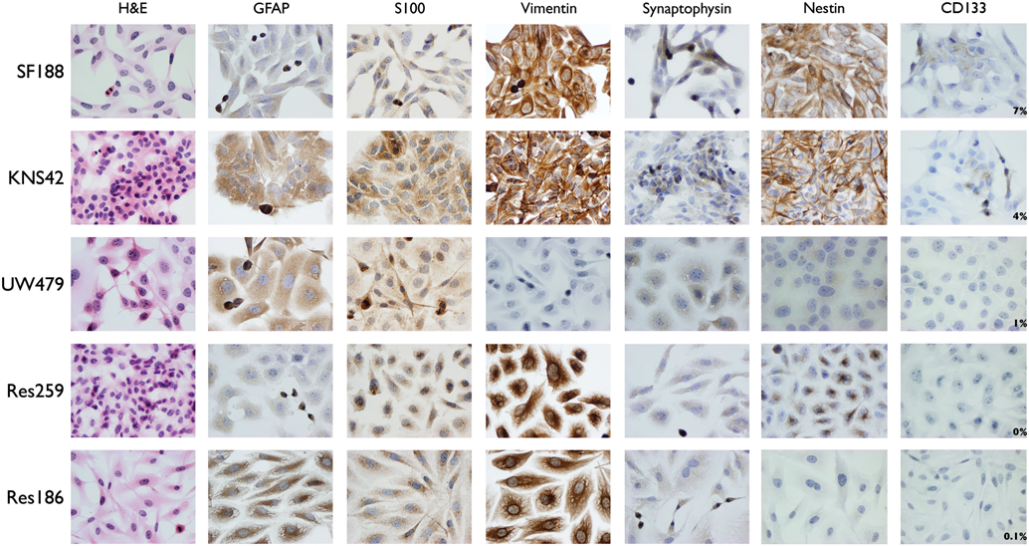
Immunophenotyping of paediatric glioma cell lines. All lines were grown as monolayers and stained with a variety of glial and stem cell markers including glial fibrillary acidic protein (GFAP), S100, vimentin, synaptophysin, nestin and CD133. H&E – haematoxylin and eosin. All images original magnification ×400.

All paediatric glioma cell lines were derived from astrocytomas of differing grades arising in patients aged 3–16 years old. They were established *in vitro* as monolayer cultures, with doubling times of between 24–48 hours, and demonstrated a mixture of stellate and bipolar morphologies, with some cells of polygonal, cuboidal or flattened appearance (Table 1). The astrocytic nature of the cells was confirmed in culture by immunohistochemistry with a range of glial markers (Figure 1). All cells showed some degree of GFAP-positivity, although SF188 and Res259 were only weakly expressing. All cells were strongly positive for S100 and vimentin, with the exception of UW479, which was vimentin negative. There was little synaptophysin expression, save for the occasional isolated cell in SF188 and KNS42 cultures. There was a surprisingly high level of expression of stem cell markers in the paediatric glioblastoma lines, with both SF188 and KNS42 strongly positive for nestin, and containing 7% and 4% CD133-positive cells respectively.

**Table 1:** Characteristics and immunophenotype of paediatric glioma cells grown as monolayers.

Cell line	WHO grade	Diagnosis	Age	Sex	Morphology	Doubling time	Immunohistochemistry					
							GFAP	S100	Vimentin	Synapto-physin	Nestin	CD133
SF188	IV	Glioblastoma multiforme	8 yrs	male	stellate, polygonal, bipolar	26 hrs	++	++	+++	++		7%
KNS42	IV	Glioblastoma multiforme	16 yrs	male	cuboidal, polygonal, bipolar	48 hrs	+++	+++	+++	++		4%
UW479	III	Anaplastic astrocytoma	13 yrs	female	polygonal, stellate	28 hrs	+++	+++	−	+		1%
Res259	II	Diffuse astrocytoma	4 yrs	female	polygonal, cuboidal, bipolar	24 hrs	+	++	+++	−		0%
Res186	I	Pilocytic astrocytoma	3 yrs	female	stellate, flattened, bipolar	46 hrs	+++	++	+++	−		0.1%

The astrocytic nature of the cells was confirmed in culture by immunohistochemistry with a range of glial and stem cell markers. (+++) strongly positive; (++) moderately positive; (+) weakly positive; (−) negative.

### Genomic profiling reveals numerous chromosomal abnormalities and focal amplifications/deletions

**Figure 2:**
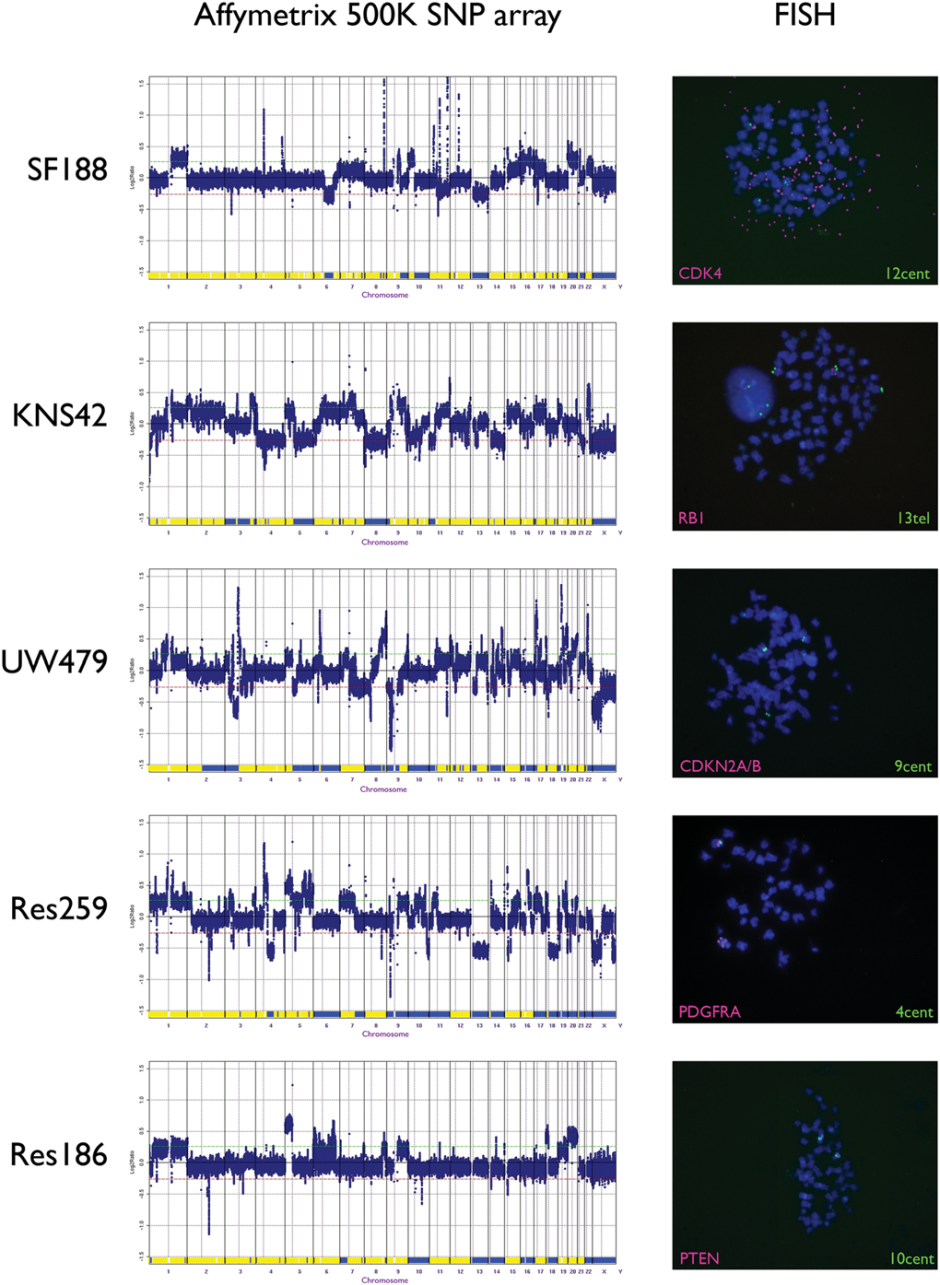
Genomic profiling of paediatric glioma cell lines. Copy number and loss of heterozygosity (LOH) profiles were generated by Affymetrix 500K SNP arrays. Log_2_ ratios are plotted (y axis) for each probeset according to chromosomal location (x axis). Loss (blue) and retention (yellow) of heterozygosity is depicted in the lower portion of the plots. Fluorescent *in situ* hybridisation validation of selected copy number changes is represented with clones for *CDK4*, *RB1*, *CDKN2A/B*, *PDGFRA* and *PTEN* (lCy5/SpectrumOrange) and appropriate centromeres (fluorescein/SpectrumGreen).

Affymetrix 500K SNP array analysis revealed all paediatric glioma cell lines to display complex genomes with numerous gross chromosomal copy number abnormalities and rearrangements. After exclusion of known copy number variants, this high-resolution copy number profiling additionally highlighted a number of focal copy number aberrations, which were confirmed by FISH on metaphase preparations (Figure 2). The glioblastoma cell line SF188 harboured high-level amplifications of several oncogenes known to be present in paediatric glioblastoma tumour samples. These included *MYC* at 8q24, *CCND1* at 11q13, *CDK4* at 12q14, all of which were due to extrachromosomal double minute formation, and overexpression of the corresponding proteins was confirmed by Western blotting (data not shown). There was furthermore a focal deletion of *NF1* at 17q11.2. KNS42 glioblastoma cells, by contrast, contained no genuine amplifications, but instead its highly rearranged genome harboured low-level copy number gains at loci such as 3q26 (*PIK3CA*), and hemizygous deletions at known tumour suppressor loci such as 13q14 (*RB1*). FISH analysis confirmed that this was due to the loss of a single copy from an otherwise triploid genome. Homozygous deletion at the *CDKN2A/B* locus at 9p12 was observed in the anaplastic astrocytoma UW479 line, which additionally harboured numerous high level copy number changes including 3p11-p12, 6p21, 18p11 and 19q12. *CDKN2A/B* deletion was also observed in Res259 astrocytoma cells, which also contained the well-described 4q12 amplicon, resulting in high level gains of the oncogenes *PDGFRA* and *KIT*, and low-level gain of a region including *KDR/VEGFR2*. This was found from FISH and M-FISH analysis to be due to an unbalanced translocation between chromosomes 4 and 19, resulting in a der(19)t(4;19). Finally, Res186, originally derived from a pilocytic astrocytoma, harboured the least complex genomic profile, although was found to have an intragenic homozygous deletion of *PTEN* at 10q23.

### Copy neutral and epigenetic events in paediatric high grade glioma cells

In addition to the copy number changes, we also observed several copy neutral loss of heterozygosity (LOH) events (Figure 2). KNS42 cells exhibited focal copy neutral LOH at 19p13 (*KLF2*) and 21q21 (*HEMK2*), whilst larger regions were observed in UW479 cells at chromosome 2q11-qter and in Res186 at 7p11-pter and 17p13 (*TP53*). Although the significance of these events is not clear, it is expected that such loci may harbour genes in which the remaining allele may harbour a point mutation, as is the case for *TP53*.

In order to explore epigenetic events in these lines we carried out methylation-specific multiplex ligation-dependent probe amplification (MS-MLPA) on 39 genes known to be hypermethylated at CpG islands in human cancer. Certain genes such as *RASSF1A* and *ASC* were found to be methylated in all cell lines tested (Table 2). There was specific promoter hypermethylation in *FHIT* in SF188 and Res259, *HIC1* in Res259 and *TP73* in Res186 cells. Interestingly, UW479 additionally harboured extensive promoter hypermethylation at a host of genes assayed including *APC*, *CASP8*, *CD44*, *CDH13*, *CHFR*, *ESR1*, *GSTP1*, *IGSF4*, *MGMT*, *PAX5A*, *PAX6*, and *RARB*. Of direct relevance to clinical resistance to alkylating agents were the observations of methylation in the mismatch repair gene *MLH3* in Res259 cells, which also harboured high levels of promoter hypermethylation of *MGMT*, along with KNS42 cells. Low to moderate *MGMT* methylation was also observed in UW479 and Res186 cells.

**Table 2:** Methylation-specific multiplex ligation-dependent probe amplification of 39 genes in paediatric glioma cell lines.

Gene	SF188	KNS42	UW479	Res259	Res186
**ASC**	52%	66%	52%	81%	100%
**APC**	2%	2%	100%	1%	2%
**ATM**	0%	2%	1%	1%	0%
**BRCA1**	1%	1%	2%	3%	3%
**BRCA2**	0%	0%	0%	0%	0%
**CASP8**	1%	3%	71%	1%	3%
**CD44**	0%	2%	48%	1%	0%
**CDH13**	2%	24%	94%	4%	0%
**CDKN1B**	0%	0%	0%	0%	0%
**CDKN2A**	2%	1%	**DEL**	**DEL**	3%
**CDKN2B**	3%	4%	**DEL**	**DEL**	4%
**CHFR**	0%	1%	81%	0%	0%
**DAPK1**	5%	0%	0%	0%	0%
**ESR1**	25%	51%	31%	74%	55%
**FHIT**	62%	0%	0%	56%	2%
**GATA5**	10%	0%	14%	86%	94%
**GSTP1**	3%	5%	75%	0%	70%
**HIC1**	2%	2%	7%	92%	0%
**IGSF4**	0%	0%	44%	0%	0%
**MGMT**	11%	79%	26%	63%	57%
**MLH1**	1%	1%	1%	1%	2%
**MLH3**	0%	0%	0%	70%	0%
**MSH2**	0%	0%	0%	0%	0%
**MSH3**	0%	0%	3%	5%	7%
**MSH6**	0%	0%	0%	7%	0%
**PAX5A**	43%	46%	99%	53%	100%
**PAX6**	0%	0%	100%	0%	0%
**PMS2**	0%	0%	3%	3%	0%
**PTEN**	4%	1%	1%	2%	**DEL**
**RARB**	5%	4%	56%	2%	0%
**RB1**	5%	0%	0%	0%	23%
**RASSF1A**	95%	90%	100%	100%	100%
**STK11**	0%	2%	0%	0%	0%
**THBS1**	5%	9%	9%	10%	0%
**TIMP3**	0%	16%	0%	1%	2%
**TP53**	1%	4%	3%	1%	22%
**TP73**	0%	0%	0%	0%	43%
**VHL**	0%	0%	0%	0%	0%
**WT1**	0%	88%	7%	69%	21%

Values give percentage methylation at CpG islands in the genes promoter. DEL = gene deletion. n.d = not done.

### Differential constitutive activation of PI3K, MAPK and downstream pathways

**Figure 3:**
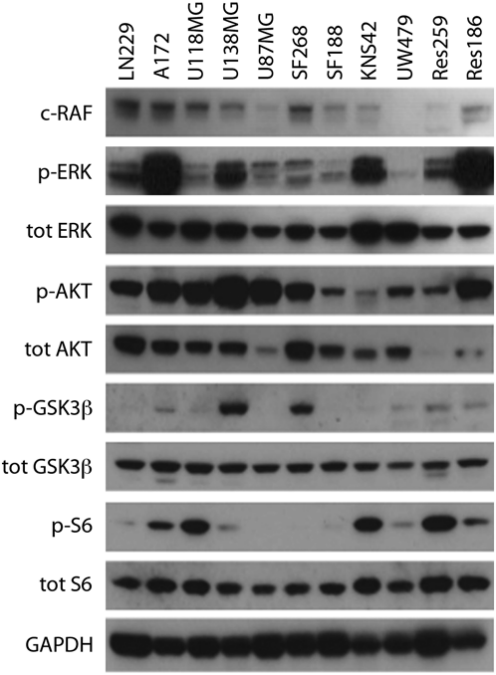
Constitutive activation of key signalling pathways in glioma cell lines. Western blots analysis of c-Raf, phospho/total Erk1/2, phospho/total Akt, phospho/total GSK3β, phospho/total S6 and GAPDH as loading control in adult (LN229, A172, U118MG, U138MG, U87MG, SF268) and paediatric (SF188, KNS42, UW479, Res259, Res186) glioma cell lines.

Protein expression was measured by Western blot to examine constitutive pathway activation of several key signalling transduction networks of importance in gliomagenesis (Figure 3). There was a modest activation of the MAPK pathway as determined by levels of phosphorylated Erk 1/2 in the paediatric high grade lines SF188 and UW479 as compared to KNS42 and the low grade lines (Res259, Res186). Although Akt pathway activation was seen in all paediatric cells, this was considerably lower than that observed in our panel of adult glioblastoma lines, except in the *PTEN* null line Res186, which showed high levels of phospho-Akt. Phosphorylated GSK3β levels were low in UW479, Res259 and Res186, and almost entirely absent in the glioblastoma lines SF188 and KNS42. Although there was some degree of ribosomal protein S6 activation in all lines, this was particularly pronounced in KNS42 and Res259, with very high levels of phospho-S6. Taken together, although variable, we observed constitutive activation of one or more of these signal transduction pathways in all the paediatric glioma lines.

### Expression signatures of functionally relevant genes distinguishes between high grade paediatric and adult glioma cell lines

**Figure 4:**
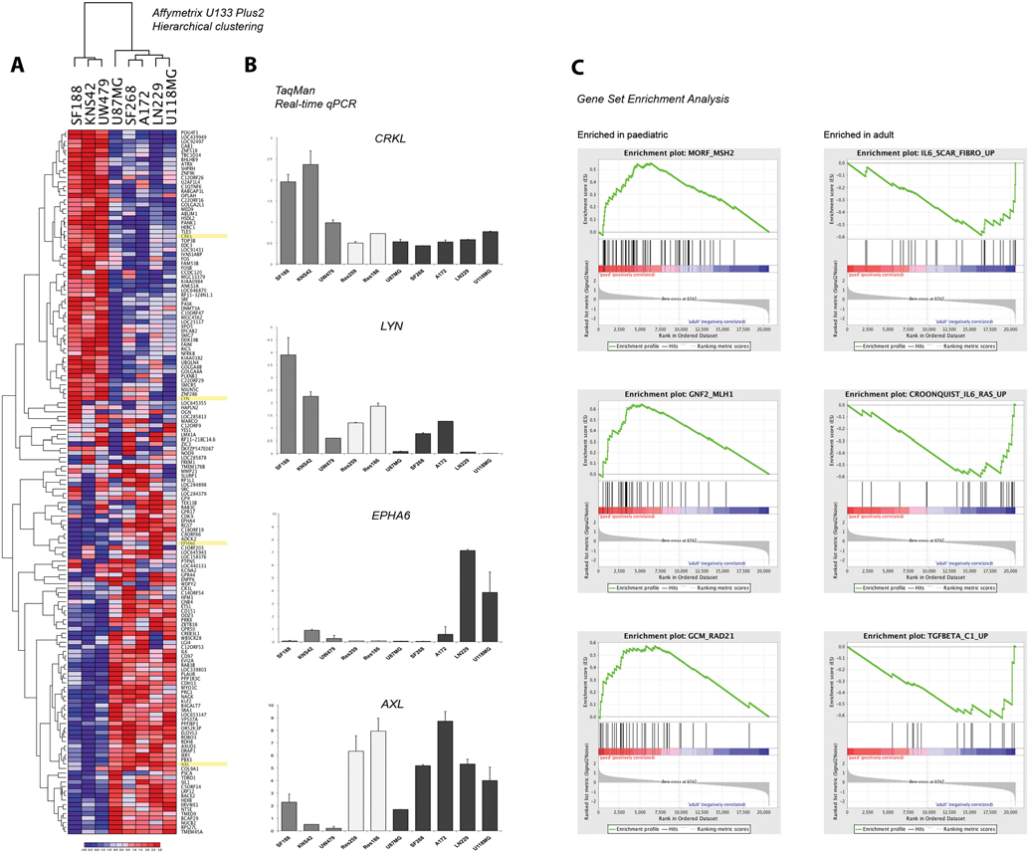
Expression profiling of paediatric and adult glioblastoma cell lines. (A) Heatmap demonstrating hierarchical clustering of 93 differentially expressed genes between paediatric (SF188, KNS42, UW479) and adult (LN229, A172, U118MG, U87MG, SF268) high grade glioma cell lines. (B) Quantitative real-time (TaqMan) RT-PCR confirming differential expression of *CRKL*, *LYN*, *EPHA6* and *AXL*. Expression values are plotted relative to Universal Human Reference RNA. (C) Gene Set Enrichment Analysis highlighting co-ordinated differential expression of gene sets defined *a priori*. Enriched in paediatric high grade glioma cell lines - MORF_MSH2, GNF2_MLH1, GCM_RAD21, DNA_replication_reactome; enriched in adult lines IL6_SCAR_FIBRO_UP, CROONQUIST_IL6_RAS_UP, TGFBETA_C1_UP. Nominal p value<0.001.

In order to investigate the differences between the paediatric- and adult-derived cell lines at the gene expression level, we carried out expression profiling using Affymetrix U133 Plus2.0 arrays. For the comparison, we excluded the lines Res259 and Res186, derived from low-grade tumours, in order to avoid the possible confounding factors of grade. Despite the small number of lines profiled, we were able to identify 93 genes significantly differentially expressed between paediatric and adult high grade glioma cell lines (Figure 4A). The differentially expressed genes included several kinases which were validated by quantitative real-time RT-PCR (Figure 4B). These included members of the Src family kinases - *LYN* and the adaptor molecule *CRKL*, highly expressed in paediatric lines, along with *SRC* itself and also *YES1*. Also upregulated in the paediatric *vs* adult lines were several early response genes including *FOS* and *FOSB*. Highly expressed in the adult lines were the receptor tyrosine kinases *EPHA6* and *AXL*, and also *CDK9* and *ILK*.

In addition, gene set enrichment analysis (GSEA) highlighted co-ordinated differentially high expression of several gene lists associated with maintenance of DNA replication and repair in paediatric *vs* adult cell lines (MORF_MSH2, GNF2_MLH1, GCM_RAD21, DNA_replication_reactome) (Figure 4C). Furthermore, gene sets representing co-ordinated regulation of extracellular matrix protein in response to cytokines were found enriched in the adult compared to the paediatric lines (IL6_SCAR_FIBRO_UP, CROONQUIST_IL6_RAS_UP, TGFBETA_C1_UP). Although the nominal p values of these analyses was less than 0.001, q values for estimates of the false discovery rate were all greater than 0.9, presumably due to the small sample size.

## Discussion

Understanding and exploiting the similarities and differences between adult and paediatric gliomas is an important strategy for the development of new targeted therapies. The intrinsic drug resistance of high grade gliomas represents a major challenge in the clinical management of children with the disease, and efforts to understand and overcome this are hampered by a lack of mechanistic data in paediatric glioma models. We have sought to address this by collecting and carrying out a detailed molecular and phenotypic characterisation of the few available paediatric glioma cell lines, with a view to incorporating them into ongoing drug development programmes.

There is an acute clinical need to develop better treatment strategies for the refractory high grade gliomas in childhood, and having lines representative of paediatric glioblastoma (WHO grade IV – SF188 and KNS42) and anaplastic astrocytoma (WHO grade III – UW479), would be extremely useful in preclinical and mechanistic studies of the disease. These lines all grew as either stellate or bipolar cells, with some polygonal features, in monolayers, and the immunophenotypes were representative of high grade glial tumours, including expression GFAP, S100 and vimentin. There were slight discrepancies with a previous publication referring to some of these lines [19], with a greater degree of S100 staining in UW479, and less GFAP in Res259 in our study, however it unclear whether this is reflective of methodological differences, culture conditions or changes in gene expression over time.

It is of considerable interest to note the relative high levels of CD133 positive cells in both SF188 (7%) and KNS42 (4%). Intrinsic drug resistance in the proportion of clinical glioblastomas comprised of tumour stem cells has been suggested to play a key role in drug resistance in this disease, and the high CD133-positive content of these cells grown as monolayers may make them particularly relevant in preclinical drug screening programmes.

All three high grade lines had highly complex genomic profiles and harboured amplifications and deletions at several known cancer genes dysregulated in paediatric glioma, including *CCND1*, *MYC*, *CDK4*, *PIK3CA*, *CDKN2A/B*, and *RB1* [2], [20]–[23]. Although there are no molecular alterations yet described that are diagnostic for paediatric high grade glioma, these genes cover a broad spectrum of reported genomic aberrations. In addition, large scale chromosomal imbalances such as gain of chromosomes 1q and 7, and losses of 10q and 13q, reported at high frequencies in primary tumours [24], are present within these cell lines.

There were contrasting genomic profiles in the two paediatric glioblastoma lines. SF188 harboured an amplifier phenotype, with coordinated amplification of *CCND1* and *CDK4* evidence of a lack of cell cycle control by virtue of dysregulation of the RB1 pathway. By contrast, KNS42 cells achieved a similar end through direct deletion of *RB1* itself. No *RB1* hypermethylation was detected. Simultaneous abrogation of core signalling pathways in glioblastomas, as recently reported for primary tumours [25], [26], are in evidence in both these cell lines, with further disruption to the p53 pathway (through point mutations of *TP53* in both lines), and the RTK/PI3K/AKT pathway via *NF1* deletion in SF188 and *PIK3CA* copy number gain in KNS42.

Interestingly, UW479 cells, as well as harbouring numerous high level amplifications, also had extensive promoter hypermethylation at a variety of epigenetically regulated genes, including *APC*, *CASP8*, *CD44*, *CDH13*, *CHFR*, *ESR1*, *GSTP1*, *IGSF4*, *MGMT*, *PAX5A*, *PAX6*, and *RARB*. They also had, in common with all the lines tested, methylated *RASSF1A* and *TMS1* genes. *RASSF1A* promoter methylation is one of the most common molecular changes in cancer, and may modulate multiple apoptotic and cell cycle checkpoint pathways [27]. *TMS1/ASC* is an intracellular signaling molecule with proposed roles in the regulation of apoptosis, nuclear factor-κB activation, and cytokine maturation, and has been found to be hypermethylated in approximately 40% of adult glioblastoma cell lines and tumours [28].

Of potential significance in preclinical drug screening was the observation that UW479 and Res186 cells to harbour methylated GSTP1. Glutathione-S-transferase π acts to enzymatically conjugate glutathione to the reactive metabolites of alkylating agents, and up-regulation in glioblastoma tumour specimens has been correlated with resistance to BCNU [29]. Of further direct translational relevance was the observation that KNS42 cells were found to have methylation in the *MGMT* promoter, but counter to the expectation in glioblastoma cells, this did not confer a sensitivity to *in vitro* treatment with the alkylating agent temozolomide [30].

Two of the cell lines (Res259 and Res186) were reported to be diffuse astrocytoma (WHO grade II) and pilocytic astrocytoma (WHO grade I), which would make them extremely valuable *in vitro* models. Due to the difficulty in culturing these lesions successfully, and the relatively stable genomes reported in the human disease [31]–[33], it is likely that these lines may have acquired genetic alterations in culture. Interestingly, both lines harboured genetic alterations reminiscent of high grade gliomas in humans - including the archetypal 4q12 amplification spanning *PDGFRA* and *KIT* in Res259, and *PTEN* deletion in Res186. Despite this, their immunophenotypes more closely resembled low grade lesions, with an absence of synaptophysin expression, in contrast to the occasional positive cells seen in the high grade lines, and higher grade tumours in humans [34]. Whether or not they are truly reflective of the lesion from which they were derived, they are likely to make excellent models for the study of specific pathway modulation, *i.e.* that of the receptor tyrosine kinase and PI3K/PTEN systems. This is of particular importance, as the paediatric high grade lines were all *PTEN* wild-type, and there was significantly lower constitutive levels of phosphorylated Akt in these cells than a selection of the commonly used adult glioblastoma lines.

Similarly, constitutive activation of additional key signalling pathways would further increase the usefulness of the cell line panel for drug development. The MAPK pathway is represented in the KNS42 glioblastoma cells, which otherwise has little constitutive activation of Akt. High levels of phosphorylated S6 in both KNS42 and Res259 cells shows a significant activation of the mTOR pathway, potentially independent of upstream PI3K/Akt signalling. mTOR is emerging as a therapeutic target in human gliomas [35], and extending this to the paediatric setting will be facilitated by the use of these models for screening mTOR inhibitors, alone and in combination strategies.

Global measures of gene expression also served to highlight the differing genetic background of the paediatric lines. The differential expression of diverse kinases including *LYN*, *CRKL*, *EPHA6* and *AXL* between the high grade glioma cells derived from patients of different ages suggests a number of potential new drug targets, and provides impetus for molecular pathology investigations to confirm their presence *in vivo*. Two such observations are the overrepresentation of genes associated with DNA integrity in the paediatric cells, and underrepresentation of those associated with the immune response. As both of these areas are under intense research for the development of novel therapeutic agents in gliomas [36]–[40], measurement of the specific elements responsible for their modulation is needed in order to ensure the most appropriate model is utilised for mechanistic and preclinical assessment.

The paediatric lines have already proven their usefulness *in vitro* with studies identifying differential drug sensitivity and mechanisms of action/resistance [19], [30], [41], [42]. As an adjunct to this, we are currently establishing and profiling them as *in vitro* neurosphere cultures, and *in vivo* as xenografts in immunodeprived mice. It is clear, however, that to fully represent the heterogeneity present in human glioma [25], and particularly those derived from children, additional well-characterised models will be required if we are to reliably test novel agents aimed at treating these diverse, clinically refractory tumours.
